# Adaptation, Validity, and Reliability of the Patient Health Questionnaire (PHQ-9) in the Kurdistan Region of Iraq

**DOI:** 10.3390/healthcare11040598

**Published:** 2023-02-16

**Authors:** Francesca Policastro, Alessandra Rossi, Hushyar Musa Sulaiman, Nezar Ismet Taib

**Affiliations:** 1 Department of Medicine and Health Science, University of Trieste, 34100 Trieste, Italy; 2 Italian Association for Solidarity Among People (AISPO) NGO, Duhok 42001, Kurdistan Region of Iraq, Iraq; 3 Duhok Directorate General of Health, Ministry of Health, Duhok 42001, Kurdistan Region of Iraq, Iraq; 4 Department of Medical Sciences, Child and Adolescent Psychiatry, Uppsala University, 75105 Uppsala, Sweden

**Keywords:** patient health questionnaire-9, validity, reliability, primary health care settings, Kurdistan region of Iraq

## Abstract

Aim: The Patient Health Questionnaire (PHQ-9) is widely used for detecting and screening depression in Iraq. However, no psychometric assessment has been performed on any Iraqi version. This study aims at studying the reliability and validity of the Iraqi Kurdish version of the PHQ-9 as tool for identifying depression. Methods: A cross-sectional study design was used; data were collected from 872 participants (49.3% female and 51.7% male) at Primary Health Care Centers (PHCCs) in the host community as well as from Internal Displaced Persons (IDPs) and refugee camps. Sociodemographic information was obtained; PHQ-9 for the diagnosis and screening of depression and Self Reporting Questionnaire 20 items (SRQ-20) for the screening of common mental illnesses were administered. Validity and reliability analyses were performed. Results: In total, 19% of the participants had a PHQ-9 total score equal to or higher than the clinical cut-off of 10 for diagnosing depressive disorder. The internal consistency of the PHQ-9 was good (Cronbach’s alpha coefficient was 0.89). Good concurrent validity for PHQ-9 compared with SRQ-20 (71%, *p* < 0.001) was found. Conclusions: The PHQ-9 demonstrates good psychometric properties and proves to be a good tool for detecting and screening depression.

## 1. Introduction

Major depressive disorder is a common, serious, and recurrent psychiatric disorder [1]. Globally, more than 264 million people are affected [2]. There is a wide difference in the prevalence of major depressive disorder in different socioeconomic contexts and countries [3]. The 12-month prevalence of major depressive disorder is estimated to be 6%, while the lifetime prevalence is between 15 and 18% [4]. Depression is also a common disabling mental illness, severely interfering with the psychosocial function and quality of life; according to World Health Organization (WHO) estimations, by 2030, depression will be the leading cause of the global disease burden [5].

It is known that major depressive disorders are frequently under-diagnosed in low–middle income countries because of a lack of mental health care services or a lack of skilled healthcare providers to diagnose them [6]. The first and only national mental health survey available for Iraq was conducted in 2006/7, the 12-month prevalence of any disorder was 13.6% [7]. In Iraq, the common mental disorder was major depressive disorder, and the 12-month prevalence and lifetime prevalence were 4% and 7.2%, respectively [8,9]. Only 2.5% of the 12-month diagnosed patients with major depressive disorder received minimal adequate treatment [10]. Depression is the main psychiatric disorder associated with suicide in Iraq [11].

Since 2003, Iraq has experienced continued instability and repeated waves of displacement. It currently hosts about 1.206.000 Internally Displaced Persons (IDPs) and about 244,000 Syrian refugees [12,13]. They live in and out of camps in extremely challenging situations; they are at risk of many health and mental health problems, in addition to the impact of the SARS-CoV-2 pandemic (COVID-19). Studies have found that the public health measures used to prevent transmission, such as quarantine and lockdown, have had negative effects on mental health and lead to an increase in the burden and symptoms of mental illnesses; depression is one of the these mental illnesses [14,15,16,17,18]. 

There are several tools to screen for depressive disorders; the Patient Health Questionnaire (PHQ-9) is widely used for this purpose [19]. The questionnaire itself is not able to replace a psychiatric diagnosis [20] but is considered a valid and useful tool for the screening of depressive disorder in several contexts [21]. In Iraq, many screening and assessment tools have been used with no previous research on their validity and reliability in this specific cultural setting [22]. Taking into account the PHQ-9, no validity and reliability tests have been performed on any of the versions used in Iraq. The aim of this study is to determine the psychometric characteristics of the Kurdish version of the PHQ-9 in order to provide a useful and appropriate tool for the screening of depressive disorder in this specific post-war context.

## 2. Methods

### 2.1. Study Design, Setting, and Participants

A cross-sectional health facility-based study was designed and conducted between January and February 2021 in Duhok Province in the Kurdistan region of Iraq. The population of Duhok consists of about 1.648.611 host community individuals [23], 338.867 IDPs, and 80.045 Syrian refugees living in more than 20 camps and out of camps. About 35% of the population is younger than 15 years of age, 61% is 16–65, and 4% is 65 or older. The literacy rate is about 75%. The public sector employs nearly half of the active work force. More than 30% of households have a monthly income less than 500,000 IQD (about 350 USD) [24]. 

The sample was recruited from the main Primary Health Care Centers (PHCCs) including family medicine centers in the host community, IDPs, and Syrian refugee camps. Before starting the administration of the questionnaire, a proportional sample size was studied, accepting a non-response rate of 10% and a margin of error of 8%. A male-to-female gender ratio of 50:50 was set based on the 2018 KRI demographic survey. Only people older than 18 years of age were included. 

The study sample consisted of 872 participants, of which 51.7% were men. The exact male-to-female ratio of 50:50 was not respected because the administration of the questionnaire depended on access to the PHCC. The mean age was 36.1 (SD = 12.5) years, and the median age was 33 year (the median age was considered as the cut-off for grouping the sample); 23.2% of the study population was illiterate; 51.5% of the participants constituted part of the host community of Duhok, while 17.1% were refugees, and 32.4% were IDPs. 

### 2.2. Measures

#### 2.2.1. Sociodemographic Questionnaire

A semi-structured questionnaire was developed to collect the sociodemographic characteristics of the participants including age, gender, place of origin, and education level.

#### 2.2.2. The Patient Health Questionnaire-9 (PHQ-9)

The PHQ-9 was developed to detect depressive disorder in the previous 2 weeks, according to the Diagnostic and Statistical Manual of mental disorders, 4th edition [25]. This tool is not able to replace a clinician’s diagnosis but can provide a “new measure of depression severity” [20]. Each of the nine items of the PHQ-9 is rated on a Likert scale of 4 points, from 0 to 3. The total score ranges from 0 to 27. Cut-offs of 5, 10, 15, and 20 represent mild, moderate, moderately severe, and severe depressive disorder [20,21]. A cut-off of 10 or greater was used in the present study as the cut-off for the identification of depression, as suggested by meta-analysis and systematic reviews [26]. The authors of the PHQ-9 affirmed that the scale can be reproduced free of charge and without fear of copyright violation; it is intended to be used by clinicians and researchers.

#### 2.2.3. The Self-Reporting Questionnaire 20 Items (SRQ-20)

The SRQ-20 is a psychiatric case-finding tool for primary health care, created by the World Health Organization to detect mental disorders in middle- and low-income countries. SRQ-20 is also useful in mental health screening in High-Income Countries (HIC). It must consist of 20 items and investigates neurotic symptoms [27]. The participants must answer each item with yes (1 point) if the symptom is present or no (0 points) if the symptom is absent. This questionnaire has been previously used in Iraq and in the Kurdistan region, and the cut-off of 8 or higher has been described as diagnostic for mental disorders [8].

### 2.3. Translation and Content Validity

The majority of the screening tools for depression have been created in HIC for their population [28]. Therefore, a transcultural translation of the PHQ-9 and SRQ-20 was independently conducted by non-medical professional translators and a psychiatrist and analyzed for the Kurdish context. Any unclear Kurdish term was modified to include common and understandable expressions for producing a final PHQ-9 version equivalent to the source, both conceptually and linguistically. As recommended by the methodological reference provided by Beaton et al. (2000), a back-translation to English was performed and checked for inconsistency with the original English text [29]. No difficulties regarding the reconciliation of the back-translated versions emerged. Then, the final version was revised and proofread by a native speaker psychiatrist and senior researcher and shared with the interviewers for pretesting. 

Seven interviewers (3 females and 4 males) were recruited and trained to support the implementation of the study with the administration of the questionnaires. A multi-disciplinary research team, composed of a psychiatrist, an epidemiologist, a neuroscientist, and a humanitarian aid worker, conducted three online lectures focusing on the purpose of the study and orientation about the questionnaires, communication skills to conduct the interviews, and role playing to practice the questionnaires’ administration with each other. Each lecture lasted about 2 h. Furthermore, the interviewers administered a convenience sample of 28 questionnaires as pretesting. Their feedback about challenges and difficult situations faced during the field-testing were discussed in a final online meeting (online meetings were used due to the COVID-19 pandemic and public health preventive measures). Nevertheless, after the pretesting, no need to correct or change any question emerged. The interviewers did not report situations or obstacles that affected the data collection in the pretest. These interviews were not included in the present study. 

The interviewers were divided into three teams, which separately attended the PHCCs under supervision. They asked the potential participants for their written informed consent. After obtaining this, the interviewers administered the PHQ-9 and the SRQ-20. During the interviews, the answers were reported on the online version of the questionnaires to directly collect the data on the chosen software. Un-answered questions, or answers that were not understood were counted as missing data. At the end of the administration period, the multi-disciplinary research team carried out a debriefing session with the interviewers.

### 2.4. Statistical Analysis

Initially, the normality of the distribution of the total PHQ and total SRQ was determined using the Shapiro–Wilk test, which yielded a significant difference (*p* < 0.001), and thus, non-parametric analyses were performed on these variables. Then, we investigated the associations between the PHQ-9 total score and the sociodemographic characteristics that are known as risk factors associated with depression. Based on results from previous studies, we expected that women, refugees, and IDPs, as well as people with a lower educational level would have higher depression levels [30,31]. For group comparisons, we conducted a Mann–Whitney U test for the binary variables (age, gender, and community) and a Kruskal–Wallis test for the multi-category education variable. In addition, to identify the sociodemographic variables with the strongest association with the PHQ-9, we conducted a multiple linear regression analysis with the p-value stepwise, where the PHQ-9 total score was the dependent variable, and the sociodemographic data were the independent variables.

Then, the internal consistency of the tool was calculated using Cronbach’s Alpha coefficient by considering the whole study population and the subgroups created by age, gender, community, and education. We also assessed the correlation between each item of the PHQ-9 and the total score using Spearman’s coefficient.

We assessed the concurrent validity of the PHQ-9 in two ways. First, we compared the SRQ-20 total score and domain scores among participants with PHQ-9 scores (using the cut-off of 10). Considering the validity of the SRQ-20 for detecting mental disorders, we expected that the SRQ-20 scores for the overall scale and for each domain would be higher for participants with PHQ-9 greater than 9. Second, we assessed the correlation between the PHQ-9 and the SRQ-20, by using Spearman’s correlation coefficient. We established the validity of the PHQ-9 in comparison to the SRQ-20.

The descriptive and statistical analyses were carried out by using R [32]; graphs were produced by using the package Rcmdr and its plug-ins. Statistical significance for all tests was determined at a *p*-value < 0.05.

### 2.5. Ethics, Consent, and Permission

Potential research participants were met in different PHCCs. They were briefed about the key elements of the research study and what their participation would contribute to. The consent process included the request to sign a written form, available in Kurdish and Arabic languages, to document the consent to voluntarily participate in the research. Considering that more than 45% of the Kurdistan region of the Iraqi population aged 6 years and older has no primary degree [33], the possibility of interviewing illiterate persons and the need to respect their decision-making were taken into account. Therefore, attention was paid to develop the consent form in plain language to limit the lack of understanding of the scope of the research. All participants consented to participate in this study.

The study received the ethical approval of the Research Ethical Committee of Duhok Directorate General of Health and University of Duhok (protocol number 11112020-5-11) and was carried out in accordance with the Declaration of Helsinki [34]. All participants provided written informed consent. 

## 3. Results

### 3.1. PHQ-9 Scores and Depression Severity

For the 872 participants, the median PHQ-9 total score was 3.00 (IQR = 1–8). A total of 519 participants (60%) had a PHQ-9 score between 0 and 4, corresponding to the absence of major depression. Further, 188 participants (21%) had a score between 5 and 10, corresponding to mild depression; 86 participants (10%) between 10 and 14 (moderate depression); 51 participants (6%) between 15 and 19 (moderately severe depression); and 28 participants (3%) higher than or equal to 2 (severe depression). A total of 165 participants (19%) had a PHQ-9 total score equal to or higher than the clinical cut-off of 10 for identifying depressive disorder. Considering the clinical importance of item number 9 of the PHQ-9 (“Thoughts that you would be better off dead, or of hurting yourself”), 759 participants (87%) answered “not at all”, 70 participants (8%) answered “several days”, 31 participants (4%) answered “more than half days”, and 12 participants (1%) answered “nearly every day”.

Table 1 relates the PHQ-9 total score to the sociodemographic characteristics of the sample grouped by the following factors: age, gender, facility, and education. A univariate group comparison between sociodemographic characteristics and the PHQ-9 total score was set up using the Mann–Whitney test for all variables except education, for which we used the Kruskal–Wallis test.

Considering the four previous sociodemographic characteristics as explanatory variables for the final multivariate linear regression analysis, only “Gender” and “Education” were included in the final and best regression model (*p*-value < 0.001, adjusted R^2^ = 0.10).

### 3.2. Internal Consistency Reliability

The internal consistency of the PHQ-9 was good for the overall PHQ-9 scores and for each of the evaluated subgroups. Cronbach’s alpha coefficient was 0.89 for the overall PHQ-9; 0.84 for men and 0.90 for women; 0.89 for participants between 18 and 32 years of age and for those older than 32 years of age; 0.87 for the host community, and 0.91 for refugees and IDPs; 0.88 for literate participants, and 0.90 for the illiterate ones.

The internal consistency between the first eight items and the total PHQ-9 score was strong (0.62–0.76) except for question 9, which was moderate (Table 2). 

### 3.3. Concurrent Validity

The PHQ-9 and SRQ-20 were positively correlated, with a Spearman’s correlation coefficient of 0.71. In addition, Table 3 shows the distribution of the SRQ-20 scores, by grouping the participants considering the PHQ-9 cut-off (Mann–Whitney test). The median SRQ-20 scores for the overall scale and for each domain were statistically higher for the participants with a PHQ-9 score greater than or equal to 10. 

## 4. Discussion

To the authors’ best knowledge, this study is the first report about the validity and reliability of the PHQ-9 as a screening instrument for depression in the Kurdistan region of Iraq. The study found good internal consistency reliability and concurrent validity. Previously, only one study has attempted to assess the validity of some psychiatric scales for the Kurdish context, including the PHQ-9, without providing any data about the validity and reliability [19]. Based on the evaluation of an expert psychiatrist, this study also had good content validity for the PHQ-9 in the Kurdish context.

A prevalence of depressive symptoms of 19% was found among participants, higher if compared to other studies in Iraq [8,10] where the prevalence was 4%. This could be due to people suffering during the COVID-19 pandemic [17,18], in addition to ISIS conflict and continued political and financial crises [35]. The univariate comparison and the multivariate final model provided important insights about the association between sociodemographic characteristics and the PHQ-9 score. According to the study findings, gender and education are factors that impacted the mental health status of the present sample. In fact, the median scores between males and females differed, similar to the scores between different educational level subgroups. In general, depression is known to be twice as common in women compared to men. This difference is referred to as the gender gap, which is more obvious in adolescents and decreases in adulthood [36], and varies among different ethnic group and nations. Our findings are consistent with those of other studies in regard to gender. In this study, age and community were not associated with the PHQ-9. Focusing on the community where we collected the data, there was no statistically significant difference between the scores of the host community and the IDPs and refugee communities. This can be explained by the fact that the three communities were all exposed to the COVID-19 pandemic and financial crisis, and, to a certain degree, to the war with ISIS. Higher rates in the host community can be explained by the suffering during many years of war, crises, instability, and exposure to transgenerational and collective traumas [37].

Considering the psychometric characteristics of the PHQ-9 for the Kurdistan region, the reliability was adequate for the overall sample (Cronbach’s alpha coefficient = 0.89) and by subgroups (Cronbach’s alpha coefficients for the subgroups ranged from 0.84 to 0.90). Spearman’s correlation coefficients from 0.70 to 0.78 showed good internal consistency between the first eight items and the PHQ-9 total score. The level of internal consistency is similar to that of the original version of the PHQ-9, with an internal consistency of 0.89 and 0.86, respectively [28]. Item 9, about thoughts of being better off dead or hurting oneself, showed a moderate consistency with the total score (Spearman’s correlation coefficient = 0.60). This is consistent with findings of other studies that measured the internal consistency of the PHQ-9 [38,39], and for this reason the PHQ-8 is recommended for public health surveys; however, it has less specificity than the PHQ-9 [40]. Suicide thoughts and attempts are still under-reported; this could be explained by the cultural and religious effects [41], stigma, and mental health literacy level [42,43]. The concurrent validity of the PHQ-9 is supported by the statistically significant association between the PHQ-9 total score and the overall SRQ-20 score and between the PHQ-9 total score and the scores of each SRQ-20 domain (anxiety and depression, cognitive area, somatic disorders). In fact, participants with a PHQ-9 greater than or equal to 10 showed significantly higher scores in the overall SRQ-20 and in each specific domain. These associations have not been assessed previously in the Kurdistan region. The concurrent validity of the PHQ-9 demonstrates that the tool is coherent in detecting mental health issues. Based on these results, the PHQ-9 appears to be a valid and reliable tool that can support clinical examination and can improve the quality of the screening and of the diagnosis of depression in the Kurdish context.

### Strengths and Limitations

To our knowledge, this is the first study to translate, adapt, and assess the diagnostic accuracy of the PHQ-9 in the Kurdish context. The study has a number of strengths including the large sample size and its representativeness and the ability to examine subgroups of participants.

Some limitations should also be considered regarding the concurrent validity. The lack of validated depression scales for the Kurdish context did not allow a comparison between the PHQ-9 and another golden standard questionnaires. The SRQ-20 was used for this purpose, even if the validation for the Kurdish context is still ongoing. Moreover, the lack of a latent variable model test and the lack of testing–retesting could be considered to be limitations for assessing the internal consistency.

## 5. Conclusions

In conclusion, the PHQ-9 demonstrated good internal consistency reliability, content, and concurrent validity for the diagnosis of depression at the PHCC level in different communities (IDPs, refugees, and host community) in the Kurdistan region. Hopefully, these findings can contribute to providing a new validated tool for the clinical and research communities in the Kurdistan region of Iraq. The need for a psychiatrist’s examination is clear, but this tool could implement the clinical assessment itself. By considering the prevalence of mental health disorders, the lack of psychiatrists and the instability of the context, tools such as the PHQ-9 are needed to make the screening easier and to facilitate and make quicker the eventual necessary care.

## Figures and Tables

**Table 1:** Association between Sociodemographic Characteristics and the PHQ-9 total score.

Factor		*n* (%)	PHQ-9 Median (IQR)	*p*-Value
Age	18–32	406 (46.5)	3 (1–7)	0.281
	>32	466 (53.5)	3 (1–8)	
Gender	Male	451 (51.7)	2 (1–5)	<0.001 ***
	Female	421 (48.3)	5 (2–10)	
Community	Host community	483 (55.4)	3 (1–8)	0.539
	IDPs/Refugees	389 (44.6)	3 (1–7)	
Education	No education	202 (23.2)	5 (2–10)	<0.001 ***
	Primary school	218 (25.0)	3 (1–7)	
	High School	259 (29.7)	3 (1–8)	
	Bachelor’s Degree	186 (21.3)	2 (0–5)	
	Masters/PhD	7 (0.8)	1 (0–4)	

*n* = number; IQR = interquartile range. Note—significance level *p* < 0.001 ***, *p* > 0.05 not significant.

**Table 2:** Internal consistency between items and the total PHQ-9 score [26].

Items	Spearman’s Correlation Coefficient	95% Confidence Interval	*p*-Value
1—Little interest or pleasure in doing things?	0.73	0.72–0.78	<0.001 ***
2—Feeling down, depressed, or hopeless?	0.76	0.76–0.81	<0.001 ***
3—Trouble falling or staying asleep, or sleeping too much?	0.74	0.74–0.80	<0.001 ***
4—Feeling tired or having little energy?	0.74	0.73–0.79	<0.001 ***
5—Poor appetite or overeating?	0.69	0.71–0.77	<0.001 ***
6—Feeling bad about yourself, or that you’re a failure, or have let yourself or your family down?	0.62	0.72–0.78	<0.001 ***
7—Trouble concentrating on things, such as reading the newspapers or watching television?	0.68	0.66–0.73	<0.001 ***
8—Moving or speaking so slowly that other people could have noticed? Or the opposite, being too fidgety or restless that you have been moving around a lot more than usual?	0.63	0.71–0.77	<0.001 ***
9—Thoughts that you would be better off dead, or hurting yourself in some way?	0.45	0.55–0.63	<0.001 ***

Note—significance level *p* < 0.001 ***, *p* > 0.05 not significant.

**Table 3:** Distribution of the SRQ-20 scores considering the presence of depressive disorder.

	PHQ-9, <10 Median (IQR)	PHQ-9, ≥10 Median (IQR)	*p*-Value
	*n* = 707	*n* = 165	
Anxiety and Depression	1 (0–2)	3 (2–4)	<0.001 ***
Cognitive	1 (0–1)	2 (1–3)	<0.001 ***
Somatic disorders	1 (0–2)	2 (1–3)	<0.001 ***
Total score SRQ-20	4 (2–8)	12 (9–14)	<0.001 ***

Note—significance level *p* < 0.001 ***, *p* > 0.05 not significant.

## Data Availability

The data presented in this study are available on request from the corresponding author.
